# Supplementary material for: Liposomes as a Nanoplatform to Improve the Delivery of Antibiotics into Staphylococcus aureus Biofilms
Source: Pharmaceutics. 2021 Mar 2;13(3):321. doi: 10.3390/pharmaceutics13030321 (PMC7999762; doi:10.3390/pharmaceutics13030321)
Supplement: Supplementary file 1 [file pharmaceutics-13-00321-s001.pdf]

pharmaceutics

MDPI

Supplementary Materials: Liposomes as a Nanoplatform to Improve the Delivery of Antibiotics into *Staphylococcus Aureus* Biofilms

Table S1. Physicochemical properties of tested antibiotics. daddy.

Antibiotics	Molecular weight (g/mol)	Log P	pKa	Ref.
**LEV**	361.7	2.10	6.24	[47,48]
**VCM**	1485.7	2.03	2.99	[49,50]
**RFB**	847.0	4.10	6.9	[51,52]

Copyright: © 2021 by the authors. Licensee MDPI, Basel, Switzerland. This article is an open access article distributed under the terms and conditions of the Creative Commons Attribution (CC BY) license (http://creativecommons.org/licenses/by/4.0/).

Table S2. Physicochemical properties of LEV-loaded liposomes.

Lipid Composition (molar ratio)	Loading capacity (µg/µmol)	I.E. (%)	Ø (µm) (P.I.)	Zeta Potential (mV)
DMPC:CHOL:SA (7:2:1)	<2	<3	0.13 (<0.1)	+18 ± 1
DMPC:DMPG (8:2)	<2	<3	0.10 (<0.1)	-17 ± 1
DMPC:SA (9.5:0.5)	<2	<3	0.13 (<0.1)	+4 ± 1

Initial lipid concentration, [Lip]i – 30 µmol/mL; Initial antibiotic concentration [LEV]i – 1 mg/mL; Loading capacity – (AB/Lip)f (µg/µmol); I.E. (%) – Incorporation Efficiency, [(LEV/Lip)f] / [(LEV/Lip)i] x 100; Ø – mean size; P.I. – polydispersity index; DMPC – dimyristoyl phosphatidyl choline; DMPG – dimyristoyl phosphatidyl glycerol; SA – stearylamine; CHOL – cholesterol; Results are expressed as mean ± SD.

Table S3. Physicochemical properties of rhodamine-labelled liposomes used in influence of lipid. composition in *S. aureus* biofilm interaction assays and in biofilm transwell model experiment.

Formulations		Loading capacity (µg/µmol)	RFB (µg) / (0.5 µmol of lipid)	I.E. (%)	Ø (µm) (P.I.)	Zeta Potential (mV)
**LIP1**	Unloaded	---		---	0.12 (<0.1)	-23 ± 1
	Loaded	8 ± 0.2	4 ± 1	101 ± 8	0.12 (<0.1)	-24 ± 3
**LIP2**	Unloaded	---		---	0.11 (<0.1)	-19 ± 1

	Loaded	13 ± 1.7	7 ± 1	75 ± 1	0.12 (<0.1)	-21 ± 2
**LIP3**	Unloaded				0.13 (<0.1)	+12 ± 1
	Loaded	11 ± 1.3	6 ± 1	57 ± 11	0.13 (<0.1)	+10 ± 1

Initial lipid concentration, [Lip]i – 30 μmol/mL; Rhodamine concentration: 0.2 mol%; (RFB/Lip)i – LIP1 = 10 nmol RFB/μmol of lipid, LIP2 = 17 nmol RFB/μmol of lipid and LIP3 = 25 nmol RFB/μmol of lipid; Loading capacity – (RFB/Lip)f (μg/μmol of lipid); I.E. (%) – Incorporation Efficiency, [(RFB/Lip)f] / [(RFB/Lip)i] x 100; Ø – mean size; P.I. – polydispersity index.

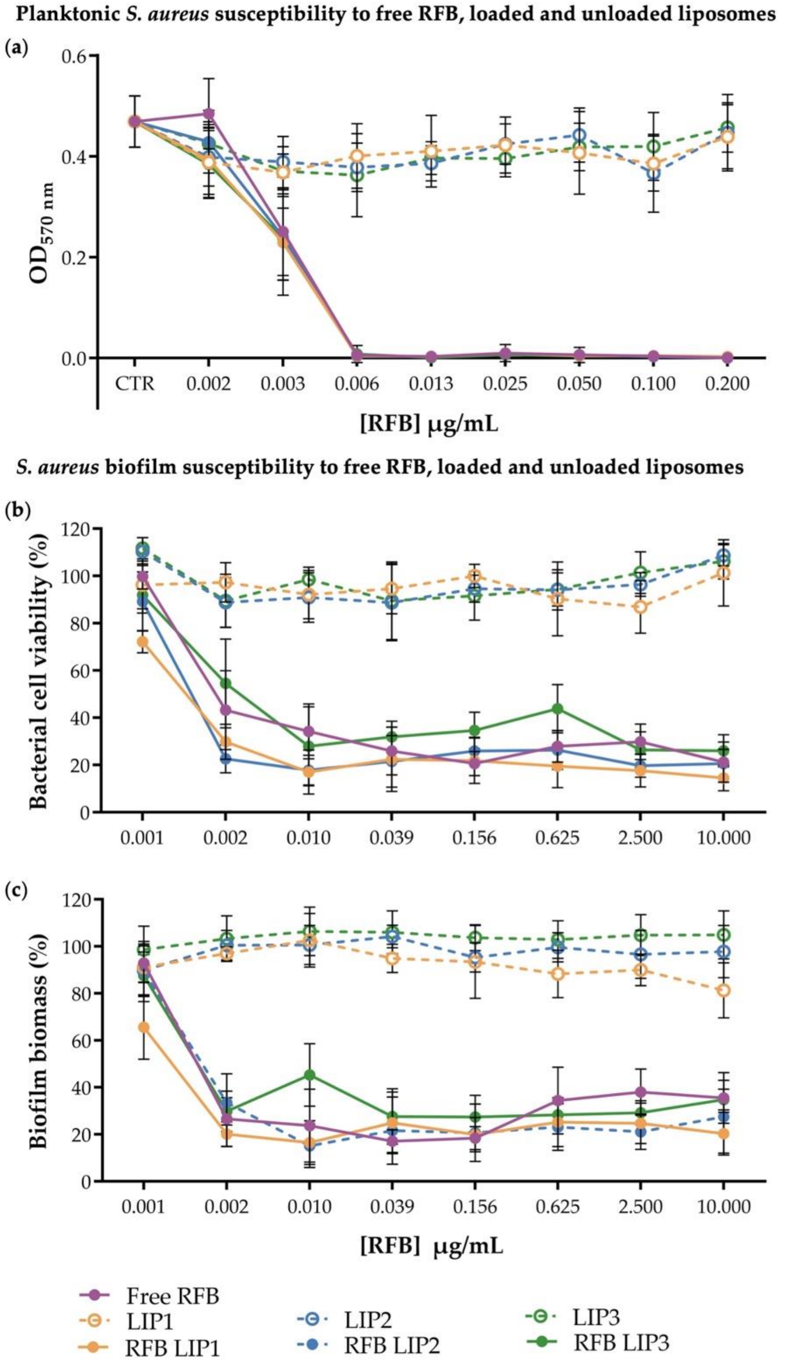

Figure S1. Susceptibility of planktonic and biofilm S. aureus to RFB-loaded liposomes. (**a**) *In vitro* planktonic MSSA susceptibility assessment through broth microdilution method followed by turbidity measurement ($OD_{570\,nm}$), after 24 h of incubation with free RFB and loaded and unloaded formulations (0.002 – 0.200 μg/mL). The positive control corresponds to untreated planktonic MSSA in MHB represented by CTR. (**b**) Determination of viable bacterial cells (MTT assay) and (**c**) biofilm biomass quantification (CV method) after performing the broth microdilution method with a RFB concentration range of 0.001 to 10.000 μg/mL, against mature MSSA biofilm. Results are expressed as mean ± SD of at least three independent experiments.

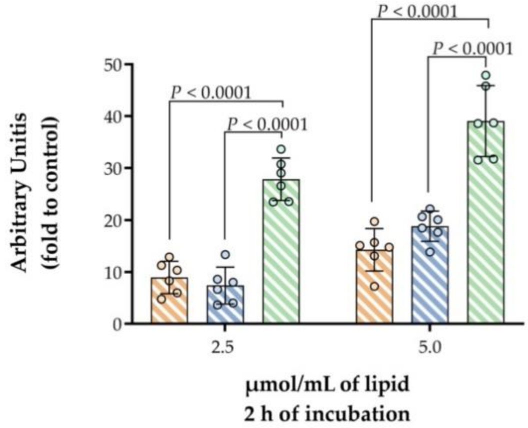

Figure S2. Influence of lipid composition on S. aureus biofilm interaction. Mature MSSA biofilm incubated with the LIP1, LIP2 and LIP3 at 2.5 and 5.0 μmol/mL of lipid for 2 h. Results are expressed as mean ± SD of at least three independent experiments. Statistical comparisons were determined by two-way ANOVA (Tukey's multiple comparisons test) analysis of variance compared between formulation groups.

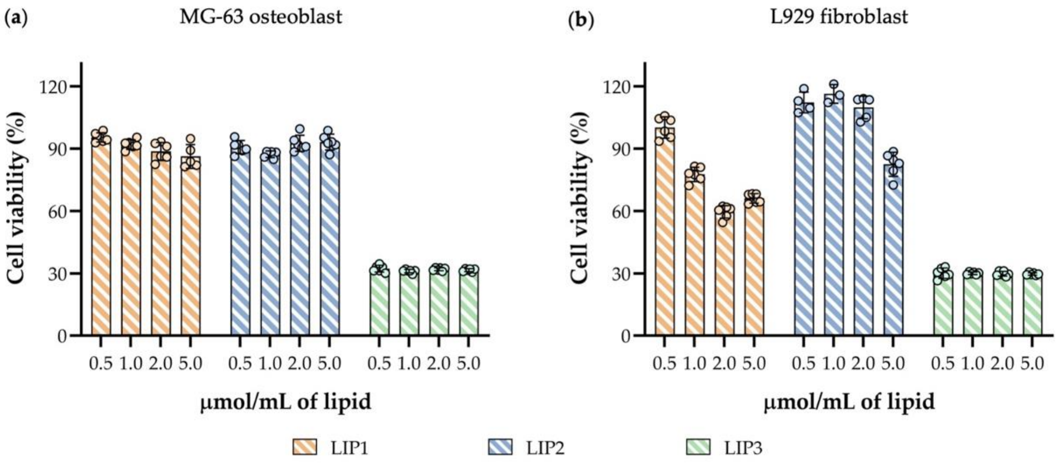

Figure S3. Cell viability of (**a**) human MG-63 osteoblast cell line and (**b**) mouse L929 fibroblast cell line, 24 h after incubation with unloaded liposomes, LIP1, LIP2 and LIP3 at lipid concentrations of 0.5, 1.0, 2.0 and 5.0 μmol/mL. Cell viability was determined by MTT reduction assay. Results are expressed as mean ± SD of at least two independent experiments.
